# Percepoes de enfermeiros sobre a assistencia ao paciente em cuidados paliativos

**DOI:** 10.15649/cuidarte.2240

**Published:** 2023-03-29

**Authors:** Roberta Brochado-da-Costa, Margarita Ana Rubin-Unicovsky, Fernando Riegel, Vagner Ferreira-do-Nascimento

**Affiliations:** 1 Enfermeira. Universidade Federal do Rio Grande do Sul (UFRGS). Porto Alegre, Brasil. E-mail: robertabc@hotmail.com Universidade Federal do Rio Grande do Sul Universidade Federal do Rio Grande do Sul Porto Alegre Brazil robertabc@hotmail.com; 2 Enfermeira, Doutora em Gerontologia Biomédica pela Pontifícia Universidade Católica do Rio Grande do Sul (PUCRS). Professora Associada do Departamento de Enfermagem Médico Cirúrgica (DEMC) da Escola de Enfermagem da Universidade Federal do Rio Grande do Sul (UFRGS). Porto Alegre, Brasil. E-mail: municovsky@hcpa.edu.br Universidade Federal do Rio Grande do Sul Universidade Federal do Rio Grande do Sul Porto Alegre Brazil municovsky@hcpa.edu.br; 3 Enfermeiro, Pós-Doutor em Enfermagem (UFSC). Doutor em Enfermagem pela Universidade Federal do Rio Grande do Sul (UFRGS). Professor Adjunto do Departamento de Enfermagem Médico Cirúrgica (DEMC) e Professor Colaborador do Programa de Pós- graduado da Escola de Enfermagem da Universidade Federal do Rio Grande do Sul (UFRGS). Porto Alegre, Brasil. E-mail: fernandoriegel85@gmail.com Autor de Correspondencia. Universidade Federal do Rio Grande do Sul Universidade Federal do Rio Grande do Sul Porto Alegre Brazil fernandoriegel85@gmail.com; 4 Enfermeiro, Doutor em Bioética (Centro Universitário Sao Camilo). Docente Assistente do Curso de Enfermagem da Universidade do Estado de Mato Grosso (UNEMAT). Campus Universitário de Tangará da Serra. Porto Alegre, Brasil. Email: vagnerschon@hotmail.com Universidade do Estado de Mato Grosso Universidade do Estado de Mato Grosso Porto Alegre Brazil vagnerschon@hotmail.com

**Keywords:** Percepgáo, Cuidados Paliativos, Enfermeiro., Perception, Palliative Care, Nurse., Percepción, Cuidados Paliativos, Enfermero.

## Abstract

**Introdujo:**

baseada em uma visáo holística do ser humano, os cuidados paliativos, tem como filosofia valorizar a vida e enxergar a morte como um processo natural.

**Objetivo:**

compreender as percepgóes de Enfermeiros na assistencia ao paciente em cuidados paliativos.

**Materiais e Método:**

estudo transversal, exploratório descritivo, com abordagem qualitativa que objetivou compreender o fenómeno do cuidado paliativo na percepgáo dos profissionais envolvidos.

**Resultados:**

Após análise dos discursos dos participantes, com base no objetivo e na semelhanga das respostas, emergiram seis categorias temáticas: Cuidado Humanizado, Lideranga, Sentimentos, Familia, Cuidado Paliativo e Conforto.

**Conclusao:**

Os profissionais apontaram como fundamental, a participagáo dos familiares e as habilidades adquiridas na experiencia vivida, a ressignificagáo diária, bem como suas limitagóes, contribuíram para compreensáo do processo de cuidar e viver humano, minimizando o sofrimento resultante de doengas terminais.

## Abstract (en)

**Introduction:**

based on a holistic view of the human being, the philosophy of palliative care is to value life and see death as a natural process.

**Objective:**

to understand the perceptions of nurses in the assistance to patients in palliative care.

**Materials and Method:**

cross-sectional, exploratory descriptive study, with a qualitative approach that aimed to understand the phenomenon of palliative care in the perception of the professionals involved.

**Results:**

After analysis of the participants' speeches, based on the objective and the similarity of the responses, six thematic categories emerged: Humanized Care, Leadership, Feelings, Family, Palliative Care and Comfort.

**Conclusion:**

The professionals pointed out as fundamental, the participation of family members and the skills acquired in the lived experience, the daily resignification, as well as their limitations, contributed to the understanding of the process of caring and human living, minimizing the suffering resulting from terminal illness.

## Resumen

**Introducción:**

basada en una visión holística del ser humano, la La filosofía de los cuidados paliativos es valorar la vida y ver la muerte como un proceso natural.

**Objetivo:**

comprender la percepciones de las enfermeras en la asistencia al paciente en paliativos cuidados paliativos.

**Materiales y métodos:**

estudio transversal, estudio exploratorio descriptivo, con un enfoque cualitativo que pretendía comprender el fenómeno de los cuidados paliativos en la percepción de de los profesionales implicados.

**Resultados:**

Tras el análisis de la discursos de los participantes, con base en el objetivo y en la similitud de las respuestas, surgieron seis categorías temáticas: Atención humanizada, Liderazgo, sentimientos, familia, cuidados paliativos y confort.

**Conclusión:**

Los profesionales señalaron como fundamental, la la participación de los familiares y las habilidades adquiridas en la experiencia vivida, la resignificación diaria diariamente, así como sus limitaciones, contribuyeron a la comprensión del proceso de Cuidar y vivir de forma humana, minimizando el sufrimiento resultante de las enfermedades terminales.

## Introdujo

O ato de cuidar é uma atividade eminentemente humana que visa promover o bem-estar do paciente fragilizado. É uma relagáo de afetividade que se configura em uma atitude de responsabilidade, atengáo, preocupado e envolvimento com o cuidador e o ser cuidado^1^.

No tocante ao cuidar, especificamente com o paciente acometido por uma patologia em estágio avanzado e sem perspectivas de cura, a atengáo e o cuidado estáo direcionados em suas necessidades e limitagóes, uma vez que o processo de morte é irreversível e o tempo de sobrevida está restrito a dias, semanas ou meses^2^.

Com base nesse entendimento, torna-se essencial adotar uma prática assistencial que esteja fundamentada no bem-estar biopsicossocial e espiritual da pessoa em sua terminalidade, a fim de proporcionar uma melhor qualidade de vida e minimizar o sofrimento durante a doenga terminal. Dessa maneira, devem-se considerar, essencialmente, os cuidados paliativos, como modalidade de assisténcia, pois, exige da equipe um olhar atento e cauteloso^3^.

Baseada em uma visáo holística do ser humano, os cuidados paliativos, tem como filosofia valorizar a vida e encarar a morte como um processo natural. Assim, náo adia, nem prolonga a morte, mas ampara o ser em suas angústias e medos, provendo o alívio da dor e de outros sintomas, oferecendo suporte para que os pacientes possam viver o mais ativamente possível, ajudando a família e os cuidadores no processo de luto^4^.

É necessário considerar, essencialmente, os cuidados paliativos, como modalidade de assisténcia, pois exige da equipe um olhar atento e cauteloso. Com base nesse entendimento, torna-se essencial adotar uma prática assistencial que esteja fundamentada no bem-estar biopsicossocial e espiritual da pessoa em sua finitude, a fim de proporcionar uma melhor qualidade de vida e minimizar o sofrimento durante a doenga terminal. A questáo norteadora deste estudo foi: Quais as percepgóes dos enfermeiros acerca da assisténcia ao paciente em cuidados paliativos? E o objetivo foi compreender as percepgóes de enfermeiros no estar com o paciente em cuidados paliativos, bem como verificar as dificuldades, limitagóes e desafios que enfrenta durante sua atuagáo.

## Materiais e Método

Trata-se de um estudo transversal exploratório descritivo com abordagem qualitativa^5^. Inicialmente, as entrevista deveriam ser realizadas na Unidade de Cuidados Paliativos do Hospital de Clínicas de Porto Alegre (HCPA), porém durante a pandemia houve o fechamento desta unidade antes da coleta de dados, frente a isso, as entrevistas foram realizadas nas Unidades do Servigo de Emergéncia (SE) do HCPA para as quais os enfermeiros foram realocados. A coleta de dados ocorreu nas seguintes unidades: Sala de Internagáo Breve (SIB) com capacidade para cinquenta pacientes, além de duas macas no box laranja para pacientes mais graves; Área COVID-19 com oito leitos e seis cadeiras para atendimento exclusivo a pacientes diagnosticados com COVID-19 e o Centro de Tratamento Intensivo, Área 1 com vinte e um leitos^6^.

Os participantes deste estudo foram cinco Enfermeiros distribuídos pelos Servigos de Enfermagem do Hospital de Clínicas de Porto Alegre (HCPA). Foram incluídos enfermeiros que atuavam na assisténcia dos Cuidados Paliativos há mais de um ano, e que concordaram em participar da pesquisa, mediante a assinatura do Termo de Consentimento Livre e Esclarecido.

Foram excluidos enfermeiros que atuavam na gestao do servido e apresentaram alguma intercorréncia no período da coleta de informagóes. Apresenta-se a seguir o Quadro 1 de caracterizado dos participantes.

**Quadro 1:** Caracterizado dos participantes do estudo. Porto Alegre, Rio Grande do Sul, Brasil, 2021.

Participantes	Sexo	Idade	Tempo de Formado Académica	Tempo de atuagáo em cuidados paliativos
Rosa	Feminino	44 anos	22 anos	6 anos
Lírio	Feminino	48 anos	25 anos	8 anos
Girassol	Feminino	39 anos	13 anos	2 anos
Cravo	Masculino	38 anos	16 anos	2 anos
Dália	Feminino	58 anos	30 anos	12 anos

Total: 5 Participantes

A coleta de informagóes foi realizada por meio de entrevista semiestruturada online via plataforma Google Meet devido a exigencia do distanciamento social decorrente da pandemia de COVID-19. A amostra inicialmente foi composta por dez enfermeiros, porém o chefe da unidade foi excluído, dois enfermeiros encontravam-se em licenga saúde, um enfermeiro em férias e outra enfermeira gozando licenga maternidade, restando cinco enfermeiros. Os questionamentos tiveram por objetivos ir ao encontro do que os participantes percebem ao estar com pacientes em Cuidados Paliativos.

As entrevistas foram gravadas e transcritas mantendo-se a fidedignidade e os significados dos discursos para posterior categorizagao e análise temática de conteúdo, buscando unidades de significado nas falas dos sujeitos que deram origem aos temas e categorias. As entrevistas foram registradas através de gravagao de mensagens em áudio, utilizando um aplicativo para bate papo. Os participantes foram representados pelos nomes de flores: rosa; cravo; lírio; girassol e dália, haja vista nao existir qualquer analogia com os seus nomes próprios.

A análise das informagóes compreendeu duas etapas. A primeira foi a caracterizagao dos participantes da pesquisa, através de dados, como, sexo, média das idades, formagao académica e tempo de atuagao junto aos pacientes em Cuidados Paliativos. Essas informagóes foram apresentadas em formas de quadros. Na segunda etapa, foi utilizada a técnica da análise de conteúdo^7^, que se estabelece em trés fases: pré-análise (organizagao das informagóes coletadas), exploragao do material (codificagao, classificagao e categorizagao dos dados) e tratamento dos resultados (inferéncia e interpretagao). Os resultados deste estudo foram analisados partindo de fundamentagao teórica que fossem alusivas a temática do estudo.

O estudo respeitou os preceitos éticos da Resolugao N^o^ 466 de 12 de dezembro de 2012. O Projeto de Pesquisa foi submetido a Comissao de Pesquisa da Escola de Enfermagem da Universidade Federal do Rio Grande do Sul, com parecer de aprovagao: 38349 e encaminhado ao Comité de Ética em Pesquisa (CEP) do Hospital de Clínicas de Porto Alegre (HCPA), e aprovado com o parecer: 3.806.502. Dos riscos e desconfortos com a realizagao da pesquisa, destaca se a identificagao dos participantes através da transcrigao os relatos, a fim de evitar este risco e desconforto foram utilizados códigos para manter o sigilo e privacidade dos participantes. Dos benefícios do estudo estao a produgao do conhecimento na área de cuidados paliativos, bem como a contribuigao para formagao de futuros enfermeiros para atuar neste contexto de cuidado.

*As* informales do estudo serao guardadas por cinco anos, mantendo a total confidencialidade dos participantes e após serao incineradas.

## Resultados e Discussao

A partir da análise das informales extraídas das entrevistas, emergiram seis categorias temáticas: Humanizado em cuidados paliativos, Lideranga em cuidados paliativos, Sentimentos, Familia, Cuidado Paliativo e Conforto.

### Humanizado em cuidados paliativos

Nesta categoria os participantes expressaram que o cuidado humanizado é o foco principal para uma assisténcia de qualidade, tendo grande importancia para aqueles que estao em contato direto a esses pacientes em cuidados paliativos. Para que os cuidados paliativos sejam integrais, e possibilitem um processo de morte humanizado, contemplando todas as necessidades básicas do paciente, sendo elas fisiológicas, psicossociais e espirituais, deve haver a participagao de uma equipe multidisciplinar na construgao desse cuidado.

O cuidado humanizado é discutido especialmente unido a Política Nacional Humanizagao (PNH) do Ministério da Saúde, com a proposta de colocar em prática um dos princípios do Sistema Único de Saúde (SUS), respeitando assim a individualidade, promovendo um ambiente confortável e acolhedor^8^.

Ressalta-se que é responsabilidade de todos os profissionais de enfermagem prestar cuidado de qualidade, mas quando o assunto é humanizagao nao podemos esquecer que os prestadores deste cuidado também sao humanos, e enfrentam paradigmas semelhantes ao do paciente quando o assunto é o processo de morte, podendo culpar a cultura ocidental que é pouco preparada para lidar com a morte^9^.

Alguns profissionais demonstraram já ter interesse na área antes mesmo de trabalharem prestando esse tipo de assisténcia, tornando assim o cuidado muito mais próximo de si, e da sua realidade, diminuindo a distancia entre o cuidado sistemático, e aproximando do cuidado humanizado e empático, como podemos visualizar no depoimento de Lírio:

*"[...] Eu sempre tive uma certa predilecto por esse tipo de paciente, porque a gente sabe que ele está nos seus últimos anos de vida em geral meses e eu acho que ele deve merecer carinho especial e eu me sinto especial dando esse retorno pra ele [...]” Lírio.*

O enfermeiro que presta cuidados paliativos deve prestar uma assisténcia que ultrapassem os limites das habilidades técnicas. Os cuidados paliativos devem ser completos e destinados a pacientes sem possibilidade de cura, ofertando desta maneira qualidade de vida para estes, que nao possuem prognostico de vida longa. Uma assisténcia embasada nas reais necessidades de cada caso e humanizada^10^.

Para os profissionais, tratar cada paciente como se fosse o primeiro é uma das várias maneiras de oportunizar um cuidado humanizado focado na sua situagao atual, desfazendo uma possível comparagao entre os casos^8^. A identificagao com os personagens está descrito a seguir:

*“[...] A minha percepgáo é de que o enfermeiro, o técnico de enfermagem também, mas o enfermeiro é fundamental nesse cuidado diferenciado e individualizado. A percepqáo que a gente tem é que nós enfermeiros temos que ser humanos no sentido de tratar cada paciente individualmente, respeitando cada um com suas especificidades, e para isso náo tem receita de bolo [...]” Girassol.*

*“[...] Eu acho que é como estar com o paciente em cuidado crítico. É como estar com o paciente em cuidados pós cirúrgicos. Cada um tem a sua característica, sua marca. Cada um é um ser único. Tém as suas particularidades [...]" Cravo.*

Considerando a singularidade dos sujeitos, há de se considerar que os enfermeiros enquanto líderes em cuidados paliativos devem buscar capacitar sua equipe para o cuidado humano e digno no fim da vida. Tal agáo exige formagáo com foco em gestáo e lideranga do cuidado.

### Lideranga em cuidados paliativos

Esta categoría analisa informagóes expressas pelos participantes de forma unanime que, o enfermeiro é um líder nato, um gestor do cuidado. Dentre as fungóes assistencias que o enfermeiro desempenha surge o papel de mentor, um educador permanente, uma referencia para tomada de decisóes para sua equipe e até mesmo para o quadro assistencial destes pacientes.

Atualmente o cenário de trabalho de enfermagem requer profissionais cada vez mais qualificados e proativos, com suas agóes aprimoradas. É esperado do enfermeiro o gerenciamento dos processos e necessidades de saúde, exercendo sua lideranga na tomada de decisóes, tendo criatividade para ser resolutivo e inovador com uma visáo ampliada^11^.

Os participantes veem o enfermeiro como precursor de decisóes, isso é identificado nas falas quando é questionado o que o enfermeiro representa:

*“[...] Tu tu é um líder, né? Chefe de equipe, uma referéncia, mesmo na equipe multiprofissional. mesmo para o médico que as vezes as pessoas pensam assim. a gente escuta ah, o paciente fala, mas eu falei com o médico, mas quem gerencia a unidade é o enfermeiro. O funcionamento da casa é o enfermeiro? como se fosse antigamente com o papel da máe, né?[...]" Rosa.*

*“[...] é o elo, de toda equipe, alipra manter um nivel alide comunicado de envolvimento, observar a delicadeza, o sentimento com que os técnicos trabalham com pacientes, suas famílias [...]" Lírio.*

*“Se percebe que o enfermeiro é líder da equipe porque passa seguranza, mantém a ordem, além de ter conhecimento de tudo [...]" Dália.*

*“[...] a importancia do papel do enfermeiro é centralizadora no sentido de que tudo deve passar por ele, né? todos os profissionais e toda a equipe de enfermagem, basicamente auxiliares e técnicos, visualizam nele o centralizador das decisoes, enfim é o comandante. Táo sempre esperando pelo enfermeiro, porque como eu disse, é necessária a presenta do enfermeiro pra dizer o que pode e o que náo pode fazer, ele é fundamental e nesse sentido é centralizado no enfermeiro e toda equipe multi também sabe que eu enfermeiro é o principal cuidador, olhador desse paciente ou destes pacientes [...]" Girassol.*

O enfermeiro procura dentro das suas possibilidades atender todas as demandas advindas do paciente, muitas vezes se tornando seu ouvinte, uma referencia em seguranza, criando vínculo, um vínculo necessário para uma assistencia de qualidade e pautada no respeito e confianza.

Liderar é a arte de influenciar pessoas, sendo assim é uma maneira de moldar como uma equipe deve trabalhar. O enfermeiro lida com situagóes imprevisíveis a todo o momento, o que leva a decisóes rápidas e coesas, influenciando diretamente na forma como conduzir o grupo, e ainda os motivar para garantir a melhoria da assistencia prestada ao paciente. No entanto, antes de exercer seu poder de lideranga, o enfermeiro deve conhecer as características do seu grupo, identificar seus talentos, dificuldades e facilidades da equipe para entao, conduzir e delegar tarefas possíveis para cada membro^12^.

*"[...] Eu acho que tá muito relacionado a parte de educando do profissional, a lideranga do Enfermeiro consiste em estabelecer maneiras de se trabalhar junto aos pacientes paliativos, acolhe-los. Também acho que deve-se acolher a equipe no sentido de auxiliá-los identificando neles sentimentos e angústias que possam interferir em relajo ao cuidado. Entao eu acho que o papel de enfermeiro junto da equipe é muito nesse sentido de ter um olhar pra esse profissional que vai um pouco além de só cuidado, só da técnica na execugao da tarefa”.[...]” Dália.*

É possível constatar nesta Categoria a importancia do papel do enfermeiro no processo de enfermagem, tornando-os administradores do servigo de saúde nas unidades que atuam, interferindo diretamente na eficácia e na qualidade da assistencia prestada.

Liderar consiste na influencia que um líder exerce sobre um grande grupo, cujo seu objetivo é influenciar positivamente, delegando fungóes ao alcance de cada membro para a nao frustragao, delimitando responsabilidade, compromisso, empatia e respeito como preceitos do trabalho a ser desenvolvido^13^.

Os enfermeiros quando indagados que sentimento está presente quando morre o paciente que estava sob seus cuidados por um longo tempo evidenciaram diversos sentimentos, deixando claro que cada profissional expressa seus sentimentos de forma distinta.

O ser humano precisa de cuidados desde antes do nascimento até o momento de sua morte. Nessa linha de pensamento, o profissional de enfermagem que lida diariamente com o processo de morte necessita de um olhar diferenciado para a fase que o paciente está atravessando, contudo, há o envolvimento deste profissional no sofrimento deste ser que é um pai, irmao, filho de outra pessoa^14^.

A equipe de enfermagem lida com o paciente constantemente, criando vínculos com ele, seus cuidadores ou familiares. O envolvimento, no caso, é algo que podemos chamar de consequencia; consequencia de um contato diário; consequencia de compartilhamento de sentimentos do paciente, do familiar para com aquele profissional^15^.

A morte é algo inevitável, mesmo mantendo o paciente com vida, fica clara a dificuldade que os profissionais tem em lidar e expressar os sentimentos relacionados ao processo de morrer^16^.

Na fala de uma das participantes observamos que os sentimentos de tristeza e alívio surgem quando o cuidado prestado a esse paciente termina:

*“[...] Eu vou dizer que, obviamente, o normal da maioria seria uma tristeza, mas nao é né? Porque tu ve uma trajetória vivida, uma etapa certa de vida e conforme as culturas, é como nós encaramos esse momento de partida. A nossa, que eu me refiro, assim é da maioria das pessoas, realmente nao é fácil de se falar em morte, mas tem algo em alguns pacientes que quase nos confortam, que estao preparados em enxergar a morte de forma tao natural que quando tu vai lá dar um abraco ou uma palavra de apoio ali, sao eles que nos confortam, né? assim como tem outros que nao, que é a maioria, mas as vezes é um alivio [...]”Rosa.*

Outra participante afirma emocionada que com o sentimento de perda de um paciente, se aprende a lidar com a opiniáo de muitos que confundem o fato de que como os profissionais lidam rotineiramente com a morte acabam se tornando insensíveis, mas no caso dela, o sentimento é de envolvimento por estar em contato com a evolugáo da doenga desse paciente, e de alívio do sofrimento dele, bem como de seus familiares que estavam em sofrimento constante. Comprovamos esta afirmagáo com a fala a seguir:

*“[...] Entao Essa questao tres é bem complicada de responder, né? Eu acho que é bem difícil assim, vou te dizer como é que eu vejo. Eu sempre ouvi falar que a pessoa com o tempo se torna insensível. Ela nao se afeta, ela tá acostumada a ver isso. Na unidade de cuidados paliativos que é uma unidade que toda hora morre pessoas é bem como foi falado na pergunta ali, quando a gente tem esses pacientes que vao pra casa, voltam, existe sim o envolvimento, um apego e nesse momento nao é tao simples. Muitas vezes a gente sai pro lado, muitas vezes a gente abraga a familia e chora junto muitas vezes a gente fica meses se lembrando das pessoas que a gente perdeu que eram pacientes, desculpa (choro)[...]” Lírio.*

*“[...] Como a gente conversa muito com a familia antes, quando ele tá naqueles dias que é a finaleira, a gente sempre deixa muito claro pra familia que apesar da pessoa partir, ela tá parando, ela tá deixando de sofrer. Entao, naquele momento a gente abraga a familia a gente diz: óhh ele foi. A gente vai sentir, mas a pessoa parou de sofrer porque nao é só um sofrimento fisico, tem falta de ar, tem o desconto da dor. É um sofrimento que as vezes a pessoa até quer que chegue aquela hora e aquela hora demora pra chegar e a pessoa quer sair daquela situagao e demora muito tempo pra acontecer. Entao as vezes eu posso afirmar que é um alivio. As vezes, vai ser um alivio as vezes pra familia que ficou envolvida que perdeu o emprego que tá numa situagao vulnerável financeiramente por tá cuidando daquela pessoa por muito tempo que tá se dedicando né?[...]” Lirio.*

Fica comprovado nas entrevistas que, surgem sentimentos ambíguos para o profissional de enfermagem que perde o paciente que, por um bom tempo estava sob seus cuidados. Afirmam que há uma falta de preparo para o sentimento de perda, mesmo que esta perda seja para alívio do sofrimento desse paciente. Pode-se demonstrar isso, conforme a fala a seguir:

*“[...] Esta questao tres, talvez seja a mais delicada com a questao da morte, porque ninguém tá preparado na nossa cultura nacional e mundial, enfim, como é lidar com a morte que, na verdade é a única coisa que a gente sabe que vai acontecer na nossa vida. Ninguém quer que isso acontega mas, a gente sabe que que vai acontecer, entao esse sentimento é complicado, ele é ambiguo porque a gente sabe que a morte vai ser tranquilizadora pra aquele paciente, porém, a gente nunca quer que a pessoa parta, né? [...]” Girassol.*

Sempre que for possível, é necessário falar, criar um espago para expor os sentimentos que nascem nos profissionais de enfermagem em relagao a morte. Visto que é de grande importancia a preservagao da saúde mental desses profissionais, que nao sabem lidar com seus sentimentos^15^.

Outra participante realga a importancia do profissional que nao se sente suficientemente preparado para lidar com a temática da dor desses familiares que acabaram de perder seu ente querido, proteger-se da maneira que melhor achar necessária, mesmo que isso acarrete o distanciamento.

*"[...] Em relajo a terceira pergunta, quando a gente tem um paciente que fica um pouco mais de tempo sobre os nossos cuidados, acabamos algumas vezes, nao é sempre que isso ocorre, criamos um certo tipo de vínculo, tu acaba virando uma referencia pros cuidados daquele paciente, mas esse cuidado traz consigo também, um leque de sentimentos. Continuando entao, o que eu sinto quando um paciente que tá há muito tempo assim sob meus cuidados e morre, isso varia muito, sabe, entao eu tento criar um certo distanciamento, mais no sentido de preservar a minha saúde mental e emocional, entao crio uma certa barreira. Cada um analisa da forma que acha mais conveniente, mais propícia [...]” Cravo.*

O enfermeiro nao é preparado durante seu curso de graduagao a lidar com os cuidados paliativos e seus desfechos, levando em consideragao toda a dor desses pacientes terminais. O profissional é preparado para promover a saúde e proporcionar melhores condigóes de vida dando conforto para o paciente e sua família. O despreparo dos profissionais por diversas vezes se torna uma barreira para descoberta dos sentimentos reais frente ao tema cuidado paliativo^17^.

Outras participantes destacam que os sentimentos que dominam sao diversos, demonstrando respeito e gratidao frente a morte.

*“[...] Ah, o sentimento que se tem no desfecho final, ele vai depender de todos os cuidados que foram norteados até o último suspiro. A gente tem que respeitar este momento único que é de muita dor para todos aqueles que com ele conviveram [...]” Girassol.*

*"[...] Com certeza, meu sentimento vai ser sempre de gratidao, de respeito frente a morte, na morte que nós entendemos que é natural. Esse empoderamento do enfermeiro em cuidados paliativos e toda equipe é quando a gente percebe que no último momento houve além do vínculo de todos, porque prestar cuidados paliativos sem haver essa relagao de vínculo, nao acontece, porque é um encontro com o outro [...]” Dália.*

Nessa diregao, enfatiza-se a importancia da família no processo de morte e morrer, buscando a compreensao da partida e do fechamento de um ciclo de vida que cada núcleo familiar vivencia de modo diferente e singular. Assim, a equipe de enfermagem deve utilizar uma lente de 360° considerando o indivíduo como um todo, prestando cuidado humano e holístico do ser em seu processo de morte.

### A importancia da participado da familia em cuidados paliativos

A referida categoria retrata a importancia do vínculo familiar e de como lidar com o sentimento da dor da perda, do sofrimento, das angústias dos familiares. A família sofre tanto ou quanto aquele paciente que está em seu processo de morrer. A partir dos relatos dos enfermeiros foi possível evidenciar como os enfermeiros administram os pequenos desejos, medos e incertezas que os familiares demandam. O enfermeiro torna-se protagonista no que se refere a gestao do cuidado no contexto dos cuidados paliativos^18^.

Para o alivio das angústias trazidas pelos familiares em algumas situagóes o enfermeiro tem que deixar o rigor técnico em segundo plano e avalia cada situagao de maneira singular para que os filhos, irmaos, pais se sintam acolhidos. O sentimento de revolta, hostilidade e negagao aparecem quando o familiar possui dificuldade de aceitagao do processo de morte. O que fica evidente no relato descrito a seguir:

*“[...] muitas vezes nos falam alguma palavra rude, algum tratamento hostil, ou até as vezes há redamares porque demorou pra atender, [...] mas ao longo do tempo tu vai sabendo que aquelas palavras, atitudes, nao foram pra ti, foram por uma revolta. Por que está acontecendo isso com a minha família? Porque eu estou aqui? Porque é que é comigo tantas vezes [...]” Rosa.*

A doenga em fase terminal pode provocar nos familiares uma variedade de reagóes emocionais, comportamentais e relacionais. Nesse sentido, a tarefa da equipe é estabelecer uma relagao de ajuda que permita aos familiares passarem por este processo sentindo que sao acompanhados. Para além de cuidados necessários a pessoa doente, os profissionais de cuidados paliativos devem também direcionar os seus esforgos com o objetivo de reforgar as capacidades e potencialidades dos pacientes e familiares, contribuindo com a restauragao da confianga dos familiares, tantas vezes perdida. Esta confianga se refere a tomada de consciencia das suas próprias capacidades (do doente/família) que irao permitir transitar por este período de vida tao sobrecarregado de experiencias agudas e assim para chegar a etapa da morte da melhor maneira possível^19^.

*“[...] Muitas vezes a familia nao sabe como lidar com a doenga terminal do ente querido, e isso suscita nela alteragóes emocionais por vezes incompreendidas na equipa. A atengao do enfermeiro deve ir de encontro as necessidades da familia, prestando-lhe informagao, apoio e educagao nos cuidados. O profissional alia-se a familia na descoberta de recursos que possam promover/devolver a estabilidade e equilíbrio familiar [...]” Dália.*

O trabalho com os familiares revela diferentes formas de prestar assistencia ao paciente, considerando as diversas fases do luto ou da morte^20^. Uma das participantes relatou que muitas vezes buscar através do diálogo alternativas para satisfazer o paciente e o familiar podem acarretar mudangas no comportamento e no relacionamento entre a equipe e a família.

*“[...] muitas vezes a gente tem que deixar um paciente que tem problemas no estomago e nao está recebendo dieta, ai o familiar reclama que ele vai morrer de fome, dai em que a enfermagem pode ajudar? O enfermeiro pode conversar com a equipe médica, e relatar a ansiedade da familia para encontrar uma solugao, dai o médico manda instalar um soro e dai explicamos ao familiar que aquilo é comida. Nada acontece com o paciente e a familiar se convence e se tranquiliza [...]” Girassol.*

Para identificar as necessidades do paciente é necessário avaliar a influencia que as intervengóes tem na qualidade de vida, desenvolvendo o manejo adequado da dor dos familiares, educando e preparando para o fim da vida^21^.

Visando amenizar a dor da partida, os enfermeiros costumam definir este momento como um descanso para o paciente e aliviar o sofrimento da família. Em outros momentos o profissional acaba tornando-se um conselheiro, dedicando momentos a reflexáo e preparando-os para o momento da despedida. O que fica evidente na descrigáo do sentimento do participante Cravo:

*“[...] acho que é uma das partes mais difíceis, pois mesmo sendo um paciente em cuidados paliativos muitas vezes quando ocorre o óbito, a família por mais que tenha o entendimento que a pessoa ia morrer logo se descontrola, grita, chora mas parece que as vezes muda tudo, e aí eles esquecem comé que seria o processo, entao o que a gente pode fazer nesse caso é acolher, ouvir e estar presente bem como, tentar oferecer algum tipo de conforto, mas eu acho que também tem que dar espado pra que eles processem isso e que eles entendam o que que aconteceu [...]” Cravo.*

No que se refere a rede de apoio antes do falecimento do ente querido, pode-se inferir que o apoio recebido pela rede influenciará diretamente em como iráo lidar com o momento da partida.

O enfrentamento desse momento de partida tem impacto diferenciado quando o familiar tem uma rede de apoio sólida, quando os profissionais acolhem as necessidades destes familiares que estáo em sofrimento vivendo o luto. O medo de encarar a realidade assombra o momento da partida desse ente querido e a dor da perda se torna imensurável, bem como o apoio psicológico é fundamental^20^. Uma das enfermeiras entrevistadas ressalta a importancia do papel do enfermeiro na educagáo e o impacto das agóes na vida desses familiares:

*“[...] O Enfermeiro diante da morte de um paciente deve estar pronto para além de enfrentar seus medos, suas dores eles devem oferecer apoio aos familiares nesse processo de luto, além de respeitar seus costumes e crengas [...]'" Dália.*

O luto é um processo longo e muitas vezes doloroso caracterizado por uma fase em que o indivíduo prefere manter-se afastado de tudo que o lembrará do motivo de sua tristeza. O enfermeiro tem a fungáo de lidar com os estágios que a morte traz a estes pacientes e seus familiares, sendo capaz de entender que náo há uma ordem correta para as fases acontecerem. É necessário que a empatia esteja presente em todos esses momentos para melhor assistir seus pacientes e seus familiares.

Para que haja um cuidado humanizado há diversos fatores a serem reconhecidos e postos em prática, um deles é a comunicagáo efetiva, que acaba se tornando uma estratégia que favorece e fortalece o vínculo criado entre a equipe e os familiares do paciente^22^. Pode-se identificar na fala de uma enfermeira entrevistada que o paciente se torna amplo, por náo ser apenas um ser com uma patologia, mas sendo também a extensáo de uma família, pessoas que sofrem junto a este paciente:

*“[...] O enfermeiro observa no contato com a família que há fatores que contribuem para o processo do luto, afetando a dinámica da familia, alterando o bem-estar físico, social, económico e emocional de todos os que convivem no contexto familiar [...]" Rosa.*

### Cuidados paliativos: necessidades a serem compreendidas no contexto de terminalidade

Esta categoria ressalta o papel da enfermagem na equipe de cuidados paliativos, na qual a identificado das necessidades emocionáis, físicas, sociais e espirituais possibilita o cuidado integral ao paciente em fase terminal.

*“[...] Esta pergunta ressalta a importancia de se prestar um eficiente cuidado integral aos pacientes terminais. Eu acho que já tá bem estruturado e fundamentados os protocolos e as diretrizes que tratam de cuidados paliativos no sentido de dar dignidade ao paciente que que tá no final da vida [...]” Cravo.*

O cuidado paliativo é considerado uma modalidade de atendimento que visa atender a todas as demandas atreladas a doenga que ameaga a continuidade da vida de um individuo. Portanto, cuidados paliativos se referem a promover qualidade de vida aos pacientes e familiares, através da prevengao e do alivio do sofrimento de paciente e seus familiares. Uma vez que este paciente requer uma avaliagao clara e focada nas suas necessidades, é preciso ir além das paredes do hospital, precisamos analisar o todo da vida deste paciente. A avaliagao e identificagao precoce da dor e outros problemas de natureza física, atrelados ao lado psicossocial e espiritual desse paciente foram o que objetivamos como cuidado integral^23^.

*“[...] Eu vejo os cuidados paliativos como um olhar especial a todo paciente terminal. Um atendimento acolhedor durante o processo que leva a morte, aliviando a sua dor e promovendo conforto. [...]” Rosa.*

Cuidados paliativos vao além da administragao de fármacos que amenizem a dor, é possível ampliar os cuidados prestados para o que o paciente acredite ser seu pilar de sustentagao, sendo ele por muitas vezes sua família, sua religiao ou crenga. É necessário saber respeitar e promover se necessário a aproximagao daquilo que traz paz ao paciente em seu processo de morte^24^.

Uma enfermeira entrevistada evidencia o sentimento do profissional quanto a lidar com esse cuidado integral, que envolve o paciente com um olhar amplo e a capacidade de atender as necessidades que este paciente traz consigo:

*“[...] é necessário ter competencia. O profissional da saúde vai desenvolvendo essas habilidades com o tempo, bem como a paciencia e o amor de encarar todas as demandas que vao vir com esse paciente, nao somente envolvendo toda a equipe mas no individual de cada profissional, mas é essa competencia que o profissional de saúde desenvolve é pra cuidar do sofrimento e a protejo a vida, enquanto houver vida [...]'" Dália.*

Outros enfermeiros entrevistados ressaltaram a importancia de prestar assisténcia qualificada e humanizada nos cuidados paliativos, destacando a necessidade de um cuidado acolhedor e personalizado.

*“[...] Eu acho assim, que toda pessoa independente do seu problema, que entra num hospital, numa UPA ou no consultório procura ajuda, ela te procura porque ela quer alguma coisa que tu possa orientar, ela quer um retorno. Entao assim, independente se é paliativo ou se nao é paliativo, eu acho que toda pessoa merece respeito, ser ouvido, acolhido, enfim, ter comprometimento [...]" Lírio.*

*“[...] A justificativa de prestar cuidados paliativos aos pacientes, eu podia te citar várias, mas eu acho que a principal é a qualidade e o conforto garantido aos pacientes e familiares nesses últimos momentos de vida, né. Entdo, assim realmente, a justificativa, ela existe plenamente [...]” Girassol.*

Uma das principáis preocupares dos profissionais de enfermagem que prestam cuidados paliativos é promover qualidade de vida e conforto para estes pacientes. A dor torna-se o sinal vital de maior importancia e todas as necessidades orgánicas desses pacientes sao essenciais. A proposta que dos cuidados paliativos é direcionada a aliviar problemas já existentes, como emocional e psicológico, bem como devolver autonima ao paciente e apoio ao familiar, mostrando que a morte é um acontecimento natural. O que demonstra um processo de morte de forma digna, sem sofrimento^25^.

O Cuidado Paliativo surge como uma nova filosofia humanitária do cuidar de pacientes em estado terminal, aliviando a sua dor, promovendo conforte e auxiliando para que o paciente sem expectativa de cura se mantenha mais ativo possível até o término de sua vida. Estes cuidados preveem a agáo de uma equipe interdisciplinar, de forma a atender o paciente terminal de forma holística, onde cada profissional deve atuar de forma a entender a importáncia de seus servidos para com o paciente em estado terminal, sobretudo, nos aspectos físicos, emocionais e espirituais^26^.

*“[...] Entdo, pra responder esta última pergunta, acho que já estou meio repetitiva mas assim, só pra reforjar, acho que as vezes o paciente puxa a campainha para se queixar de dor, falta de ar, né dai a gente tenta sanar aquilo e faz medicando, eleva cabeceira, deixa confortável, coloca oxigénio, óculos nasal, dependendo da saturando máscara de Hudson. Mas as vezes ele toca a campainha informando dor, falta de ar, mas o que ele quer mesmo é a tua presenta, teu afeto. Ai quando tu chega lá, ele quer só ateneo, conversar. Ele quer que tu pergunte como é que ele tá pra ele poder expor alguma coisa, abrir o seu corado [...]"Lirio.*

Na mesma temática que o conforto pode ser ofertado por uma escuta ativa, temos o depoimento de um enfermeiro, reafirmando que as relagóes criadas com aquele paciente ou aquele familiar, torna efetiva a assisténcia humanizada, de qualidade.

*“[...] a questdo da comunicando assertiva adequada, muitas vezes uma comunicando silenciosa, empática, mas é por meio dela que nós podemos reconhecer e acolher empaticamente as necessidades do paciente, bem como dos seus familiares [...]" Cravo.*

É necessário desenvolver agóes educativas com os familiares, promovendo o entendimento das necessidades irrefutáveis destes pacientes. É necessário que se faga entender que nesse momento é necessário ofertar para seu ente querido momentos agradáveis, lhes proporcionar boas lembrangas^27^.

O enfermeiro sempre será o mediador entre o lado emocional e racional dos familiares que estáo sofrendo junto aos pacientes:

*“[...] Os cuidados essenciais a serem prestados aos nossos pacientes é, obviamente, o controle da dor que é realmente o fator mais estressante, pros pacientes, né? Fazer com que o familiar entenda que administraruma dieta que fazia o paciente comer, nao é mais fundamental, isso é um trabalho de formiguinha que a gente tem com eles porque eles, como eu disse antes, eles querem muito que o paciente coma, se alimente e nao é isso que é prioridade para o paciente [...]” Girassol.*

Romper paradigmas que estruturam o processo de enfermagem, também se torna necessário muitas vezes para proporcionar a esse paciente um momento de prazer.

*“[...] na minha opiniao essa questao de tentar fazer algumas últimas vontades, que as pessoas tém alguns desejos, muitos querem ver parentes mais distantes. Entao se aguarda que essas pessoas cheguem, sabe pra daqui a pouco tomar algum tipo de conduta, né? mas é tudo pra pessoa ficar mais tranquila[...]” Girassol.*

*A* assistencia prestada pela equipe de enfermagem para pacientes que se encontram sem possibilidade de cura deve ser ofertada de maneira ampla, buscando atender as necessidades básicas do ser humano com empatia, respeito e competencia, com a intengáo de promover dignidade e conforto, bem-estar físico, emocional, psicológico e social. O termo conforto traz consigo os primeiros princípios de enfermagem descritos por Florence Nightingale.

## Conclusao

O enfermeiro possui papel fundamental na equipe de cuidados paliativos, especialmente na garantia do conforto para o doente terminal, considerando que estes pacientes ficam longos períodos de hospitalizados e experienciam uma variedade de sintomas, desde a constipado intestinal até a depreensáo nervosa. A atuagáo do enfermeiro inicia com a avaliagáo do grau de dependencia para o autocuidado secundário a enfermidade, ao tratamento e a reagáo do paciente frente aos desafios e problemas. Sua meta será promover maior independencia do doente, ou, quando isso náo for possível, procurar a melhor maneira de adaptado as limitagóes impostas pelo progresso da doenga. Os complexos problemas que emergem na ocasiáo dos cuidados no fim da vida, colocam-nos diante da necessidade de aprofundar o debate acerca deste momento crítico da existencia humana.

Das limitagóes deste estudo, está o fechamento da Unidade Álvaro Alvim do HCPA por motivos administrativos durante a pandemia, exigindo realocagóes de colaboradores referencia em cuidados paliativos, resultando em redugáo do número de profissionais entrevistados.

O estudo permitiu desvelar enfoques latentes na assistencia em cuidados paliativos, evidenciando a importancia da atuagáo do enfermeiro diante do processo de morte, considerando a lacuna na formagáo dos enfermeiros em relagáo ao preparo para enfrentar este desafio, pois cuidar no fim da vida exige preparo e este deve ser iniciado e aprimorado ao longo de todo o percurso formativo do enfermeiro.
